# Our Thanks

**Published:** 1872-07

**Authors:** 


### PART III.

### Editorials, Correspondence and Miscellaneous.

### OUR THANKS.

We thank our friends for the articles they have furnished. All articles will appear in order of arrival. Do not fail to write, friends. Overhaul your case-books and send us your experiences. Our second part is open to all. Fill it, friends, with the good things you have tested and can recommend to your brethren. We trust our readers will be up and doing, and aid us by pen and personal effort in giving useful and practical information to the profession.

